# Celastrol Induces Cell Apoptosis and Inhibits the Expression of the AML1-ETO/C-KIT Oncoprotein in t(8;21) Leukemia

**DOI:** 10.3390/molecules21050574

**Published:** 2016-04-30

**Authors:** Xianjun Yu, Xuzhi Ruan, Jingxuan Zhang, Qun Zhao

**Affiliations:** 1 School of Basic Medical Sciences, Hubei University of Medicine, Shiyan 442000, China; ruanxuzhi@163.com (X.R.); zhangjx@hbmu.edu.cn (J.Z.); 2 Key Laboratory of Nutrition and Metabolism, Institute for Nutritional Sciences, Shanghai Institutes for Biological Sciences, Graduate School of the Chinese Academy of Sciences, Chinese Academy of Sciences, Shanghai 200031, China; 3 Laboratory of Chinese Herbal Pharmacology, Oncology Center, Renmin Hospital, Hubei University of Medicine, Shiyan 442000, China

**Keywords:** celastrol, t(8;21) leukemia, apoptosis, mitochondrial dysfunction, C-KIT

## Abstract

Resistance to chemotherapy is a major challenge to improving overall survival in Acute Myeloid Leukemia (AML). Therefore, the development of innovative therapies and the identification of more novel agents for AML are urgently needed. Celastrol, a compound extracted from the Chinese herb *Tripterygium wilfordii* Hook, exerts anticancer activity. We investigated the effect of celastrol in the t(8;21) AML cell lines Kasumi-1 and SKNO-1. We demonstrated that inhibition of cell proliferation activated caspases and disrupted mitochondrial function. In addition, we found that celastrol downregulated the AML1-ETO fusion protein, therefore downregulating C-KIT kinases and inhibiting AKT, STAT3 and Erk1/2. These findings provide clear evidence that celastrol might provide clinical benefits to patients with t(8;21) leukemia.

## 1. Introduction

Acute myeloid leukemia (AML), a heterogeneous clonal disorder of hematopoietic progenitors, is associated with an extremely poor prognosis and an overall survival rate of only 40%–50% in adults (<60 years) [1,2]. Frequently observed chromosomal rearrangements and gene mutations play a pivotal and causal role in the induction of leukemogenesis and the progression of leukemia [3]. The t(8;21)(q22;q22) translocation is the most frequent genetic lesion observed in AML patients, with a prevalence ranging from 12% to 20% of cases [4]. The AML1-ETO fusion protein resulting from this translocation recruits cellular factors that enhance hematopoietic stem cell self-renewal, inhibit hematopoietic differentiation and promote cell proliferation, phenomena directly associated with the development of AML [5]. Previous studies have shown that the AML1-ETO fusion protein represents the first genetic hit in myeloid leukemogenesis, and point mutation in C-KIT tyrosine kinase receptor might represent the second hit [6]. Gain-of-function mutations in C-KIT are observed in 20% of AML cases, and these mutations lead to the activation of signaling pathways downstream of C-KIT, including the pathways mediated by AKT, STAT3 and MAPK [7]. We previously reported that oridonin and bortezomib might target the AML1-ETO and C-KIT oncoproteins and markedly improve the clinical outcomes in AML patients [8,9]. Several small molecule agents against t(8;21) AML cells have also been demonstrated promising results in AML cell lines [10,11]. Research efforts such as these facilitate the identification of targeted agents that can effectively treat AML. 

Celastrol is a quinone triterpene compound isolated from *Tripterygium wilfordii* Hook. F. Previous studies have shown that celastrol has multiple biological activities, including the anti-oxidant and anti-inflammatory responses [12]. Celastrol is also reported to exert anti-tumor activity. The anti-tumor effects of celastrol have been attributed to its ability to inhibit cell proliferation, induce apoptosis, and suppress invasion and angiogenesis, including lung cancer, breast cancer, prostate cancer, melanoma and glioma, *in vitro* and *in vivo* [13,14,15,16,17,18]. However, the effect of celastrol in t(8;21) AML remains unclear.

In this study, we found that celastrol inhibited the growth of the Kasumi-1 t(8;21) AML cell line by inducing mitochondrial instability and activating caspases. In addition, celastrol downregulated AML1-ETO/C-KIT and downstream signaling proteins. These results indicate that celastrol is a potential therapeutic agent for patients with t(8;21) AML.

## 2. Results

### 2.1. Celastrol Inhibits Growth and Proliferation in t(8;21) Leukemia Cells

**Figure 1:**
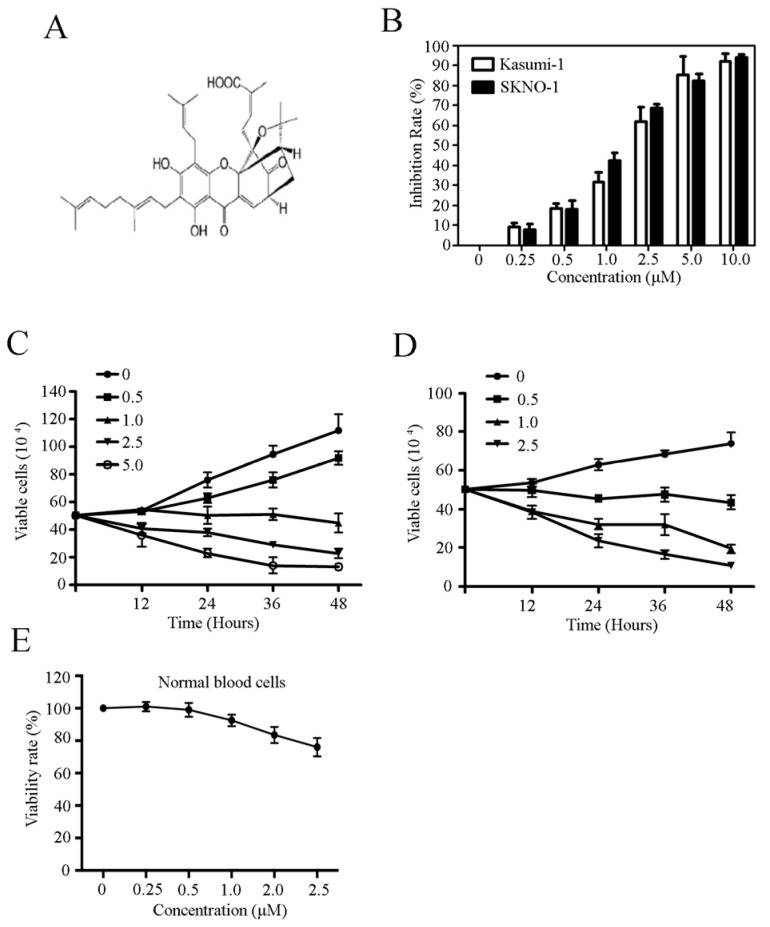
Celastrol inhibits cell growth and proliferation in t(8:21) leukemia cells. (**A**) The chemical structure of celastrol; (**B**) The growth of Kasumi-1 and SKNO-1 cells treated with celastrol for 24 h at the indicated concentration; (**C**,**D**) The effects of celastrol on Kasumi-1 and SKNO-1 cells growth as determined using trypan blue exclusion analysis; (**E**) The effect of celastrol on the growth of normal human hematopoietic cells.

The chemical structure of celastrol is shown in Figure 1A. We investigated the cytotoxic effects of celastrol on the growth of Kasumi-1 and SKNO-1 t(8;21) cell lines by treating the cells with increasing concentrations of celastrol for 24 h. As shown in Figure 1B, celastrol significantly inhibited cell growth in a dose-dependent manner in Kasumi-1 and SKNO-1 cells with an IC_50_ of 2.2 μM and 1.3 μM, respectively. We further explored the kinetics of the capacity of celastrol-induced cell growth inhibition in two cell lines and observed a dose- and time-dependent induction of cell death in cells treated with celastrol and subsequently stained with trypan blue (Figure 1C,D). We tested the effects of celastrol on normal hematopoietic cells from healthy donors, and found that normal hematopoietic cells were less sensitive to celastrol (Figure 1E). These results indicate that celastrol exhibits potent anti-leukemia activity *in vitro*.

### 2.2. Celastrol Induces Cell Death in Kasumi-1 Cells

**Figure 2:**
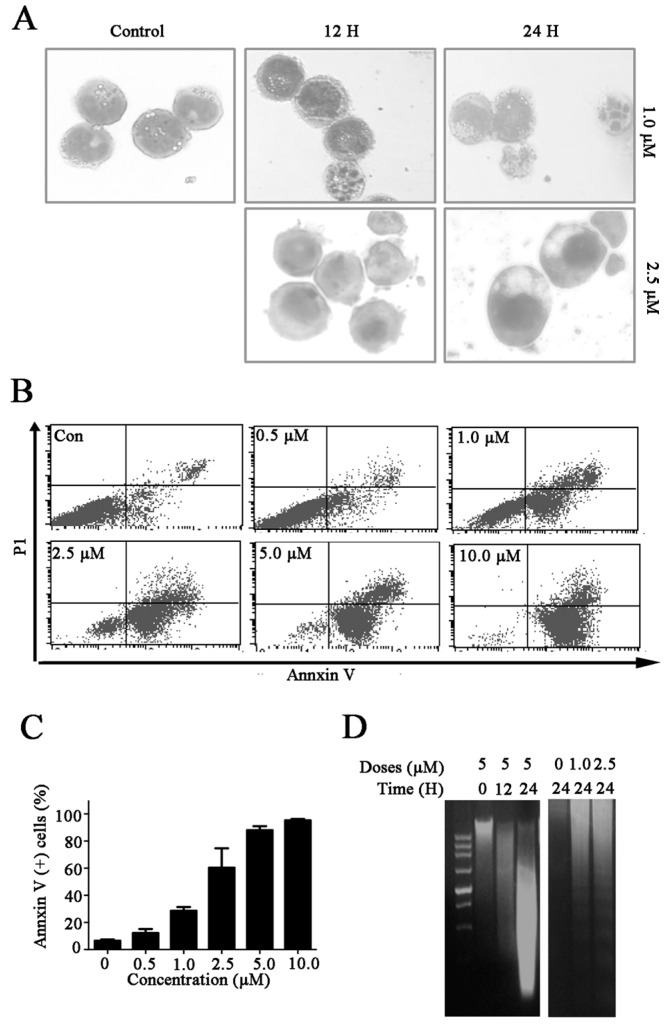
Celastrol induces cell death in Kasumi-1 cells. (**A**) Morphological characteristics of Kasumi-1 cells were treated with the indicated concentrations of celastrol for different periods of time, the changes in morphology were subsequently analyzed; (**B**,**C**) Celastrol induced cell apoptosis in Kasumi-1 cells. Cells were treated with the indicated concentrations of celastrol for 24 h, and apoptosis was analyzed using Annexin V-FITC/PI double staining and flow cytometry; (**D**) Celastrol induced DNA fragmentation. Kasumi-1 cells were treated with various concentrations of celastrol for the indicated time periods, and purified DNA for DNA fragmentation analysis.

To confirm if the cytotoxic effects of celastrol were mediated by apoptosis pathway, we first examined the cell morphology of celastrol treated Kasumi-1 cells. We indeed found that Kasumi-1 cells exhibited morphological changes after celastrol treatment, such as cell shrinkage, chromatin condensation and membrane blebbing (Figure 2A). In addition, Annexin V-FITC/propidium iodide (PI) staining revealed that the proportion of Annexin V-positive cells significantly increased from 8.21% to 86.22% in Kasumi-1 cells treated with celastrol in a dose-dependent manner (Figure 2B,C). The results from DNA fragmentation ladder assays also confirmed that treatment of celastrol increased cell death in Kasumi-1 cells (Figure 2D). Taken together, these data suggest that celastrol induces cell death in Kasumi-1 cells.

### 2.3. Celastrol Triggers Caspase Activation in Kasumi-1 Cells

**Figure 3:**
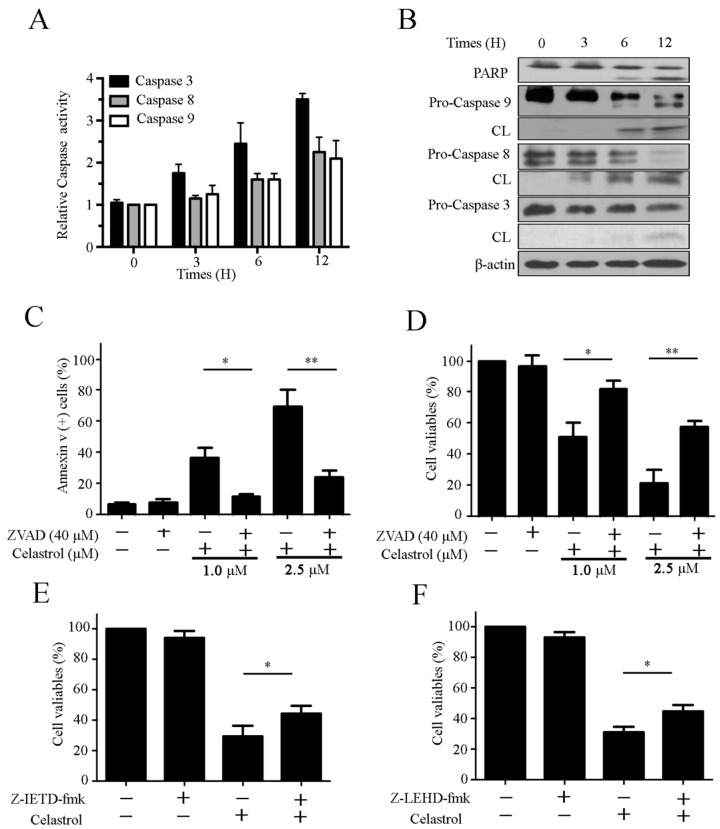
Celastrol triggers caspase activation in Kasumi-1 cells. (**A**) Caspase activity assays using Kasumi-1 cells treated with celastrol (2.5 μM) for different periods of time; (**B**) Celastrol induced PARP cleavage and activated caspase-3, -8 and-9. Kasumi-1 cells were treated with 2.5 μM celastrol for various time periods, and the cell lysates were subjected to Western blot using the indicated antibodies; (**C**) Celastrol induced cell apoptosis in a caspase-dependent manner. Kasumi-1 cells were preincubated with 40 μM Z-VAD-fmk for 2 h prior to treatment with 2.5 μM celastrol for another 24 h. Cell apoptosis was analyzed using Annexin V-FITC/PI staining; (**D**) Celastrol decreased cell viability in Kasumi-1 cells in a caspase-dependent manner. Kasumi-1 cells were preincubated with 40 μM Z-VAD-fmk for 2 h prior to treatment with 2.5 μM celastrol for another 24 h, and cell viabilities were analyzed by CCK-8 assay; (**E**,**F**) Kasumi-1 cells were incubated with 20 μM caspase-8 inhibitor (Z-IETD-fmk) (**E**) or 40 μM caspase-9 inhibitor (Z-LEHD-fmk), then treated with 2.5 μM celastrol for 24 h. Cell viabilities were analyzed using CCK-8 assays. Data are from three independent experiments. * *p* < 0.05, and ** *p* < 0.01.

To determine whether celastrol induced cell death by activating caspase, we conducted caspase activity assays. As shown in Figure 3A, celastrol significantly increased the activation of caspase-3, -8 and -9. Consistent with these, we also observed an increase in PARP cleavage (Figure 3B). To further confirm these findings, we examined the role of caspases in celastrol-induced apoptosis using the pan-caspase inhibitor Z-VAD-fmk, the caspase-8 inhibitor Z-IETD-fmk and the caspase-9 inhibitor Z-LEHD-fmk. We found that celastrol-induced cell apoptosis was suppressed by Z-VAD-fmk (Figure 3C). Z-VAD-fmk also suppressed the the celastrol-induced inhibition of cell proliferation in Kasumi-1 cells (Figure 3D). However, Z-IETD-fmk and Z-LEHD-fmk exerted only modest inhibitory effects on celastrol-induced cell growth suppression (Figure 3E,F). Together, these data imply that celastrol triggers cell apoptosis via caspase activation.

### 2.4. Celastrol Induces Apoptosis via the Extrinsic and Intrinsic Pathways

**Figure 4:**
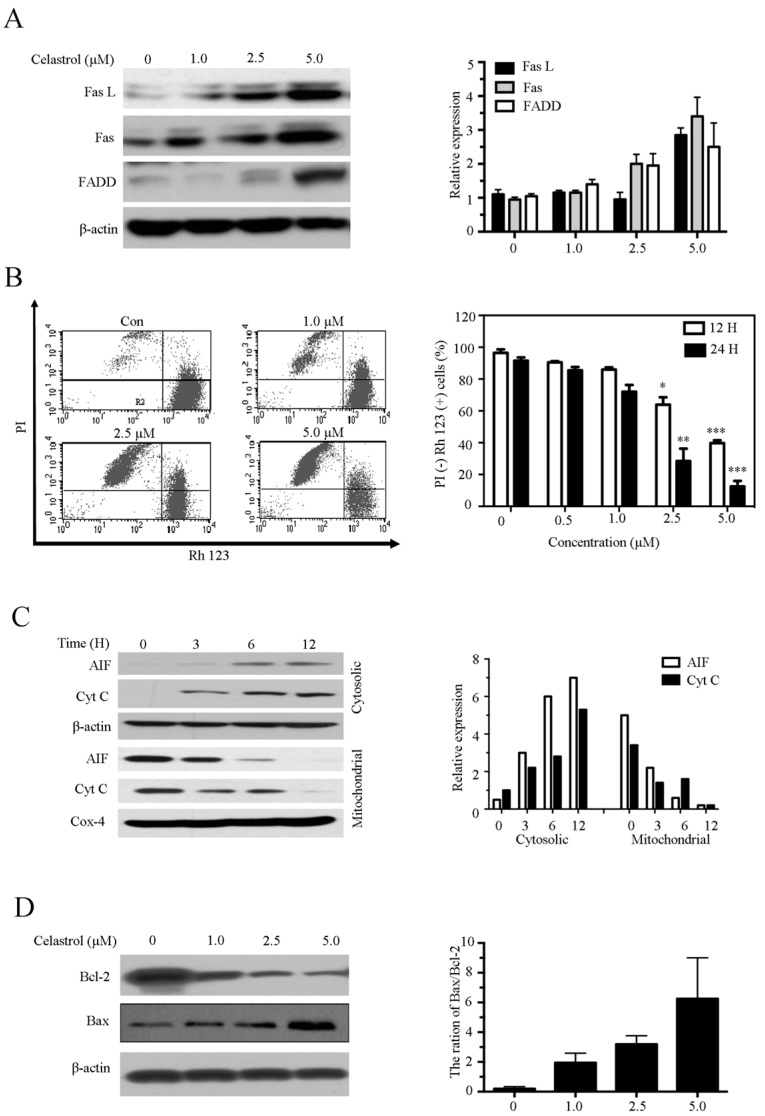
Celastrol induces apoptosis via the extrinsic and intrinsic pathway in Kasumi-1 cells. (**A**) Kasumi-1 cells were treated with various concentrations of celastrol for 24 h, and the levels of Fas, FasL and FADD were determined using western blot; (**B**) Celastrol downregulated the MMP. Kasumi-1 cells were treated with the indicated concentration of celastrol for 12 or 24 h, and the MMP was evaluated using rhodamine-123 PI staining and flow cytometry; (**C**) Celastrol induced release of AIF and Cyt C. Kasumi-1 cells were treated with 2.5 μM celastrol for 3, 6 or 12 h. Then, the cytosolic and mitochondrial fractions were separated and evaluated using western blot; (**D**) Celastrol modulated the expression of Bcl-2 family proteins in Kasumi-1 cells. Cells were treated with various doses of celastrol for 24 h, the expression of Bcl-2 and Bax were assayed using western blot. The ratio of Bax/Bcl-2 expression was analyzed. Data are from three independent experiments. * *p* < 0.05, ** *p* < 0.01, and *** *p* < 0.001.

Both the extrinsic and intrinsic apoptosis pathways trigger the activation of caspases and cell apoptosis. Thus, we sought to determine whether these pathways mediated celastrol-induced apoptosis. As shown in Figure 4A, celastrol upregulated the expression of extrinsic proteins Fas, FasL, and FADD, proteins associated with the extrinsic pathway. Next, we investigated the effect of celastrol on the intrinsic apoptosis pathways. We observed that the mitochondrial membrane potential (MMP) markedly decreased in cells treated with celastrol in a dose- and time- dependent manner (Figure 4B). An increase in the release of apoptosis-inducing factor (AIF) and cytochrome C (Cyt C) from the mitochondria into cytoplasm is a feature associated with the intrinsic apoptosis pathway. We found that celastrol potently enhanced the release of AIF and Cyt C from the mitochondria into the cytosol (Figure 4C). The intrinsic mitochondrion-mediated apoptotic pathway is also regulated by Bcl-2 family members. To determine whether celastrol disrupted mitochondria via affecting Bcl-2 family proteins, the expression of anti-apoptotic proteins Bcl-2 and anti-apoptotic proteins Bcl-2 were detected. Our results showed that celastrol upregulated Bax/Bcl-2 ratio in dose-dependent manner (Figure 4D). These results demonstrate that celastrol induces apoptosis via both the extrinsic and intrinsic pathways.

### 2.5. Celastrol Regulates the C-KIT/AML-ETO Oncoprotein and Downstream Signaling Pathway

**Figure 5:**
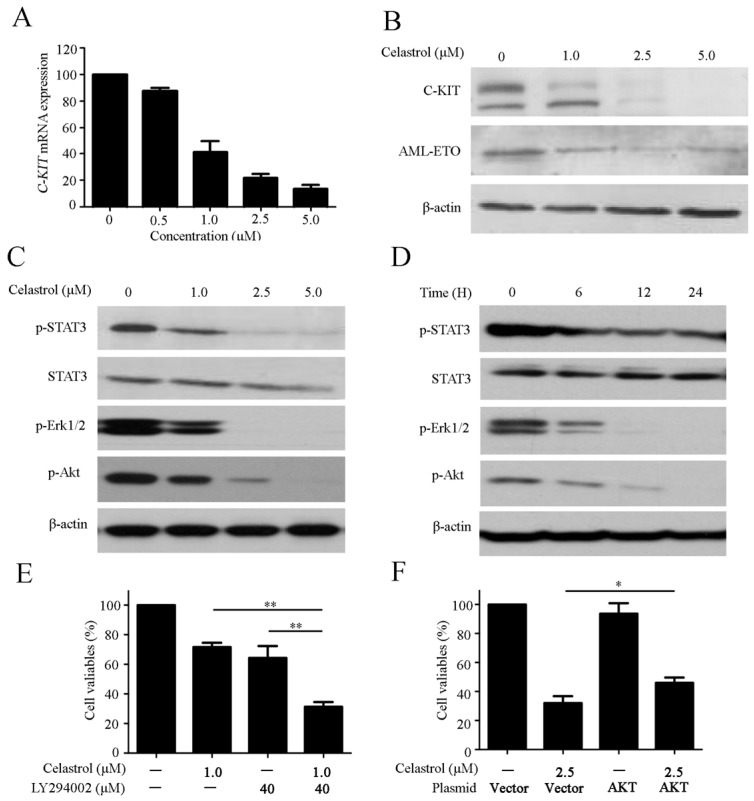
Celastrol regulates the expression of the C-KIT/AML-ETO oncoproteins and downstream signaling pathways. (**A**) The expression of C-KIT in Kasumi-1 cells treated with celastrol for 24 h was analyzed using real-time quantitative RT-PCR; (**B**) Celastrol downregulated the C-KIT and AML1-ETO protein levels; (**C**,**D**) Effects of celastrol on AKT/STAT3/Erk pathway in Kasumi-1 cells. Cells were treated with celastrol as indicated, and the expression of kinases were evaluated using western blotting; (**E**) Kasumi-1 cells were incubated with 40 μM LY294002 and 1.0 μM celastrol for 24 h. Cell viability was analyzed using the CCK-8 assay. (**F**) Kasumi-1 cells were transiently transfected with constitutively active AKT plasmid (AKT) and vector control (V) by electroporation. After 24 h, cells were exposed to celastrol (2.5 μM) for another 24 h. Cell viability was analyzed using the CCK-8 assay. Data are from three independent experiments. * *p* < 0.05, and ** *p* < 0.01.

C-KIT is a cytoplasmic receptor tyrosine kinase that plays a crucial role in the development of t(8:21) AML [6]. We then examined the effect of celastrol on C-KIT. We indeed observed that treatment of Kasumi-1 cells with celastrol effectively inhibited the expression of C-KIT mRNA (Figure 5A). In addition, western blotting also demonstrated a dramatic reduction of the C-KIT and AML1-ETO protein levels in Kasumi-1 cells treated with celastrol (Figure 5B). The AKT-, signal transducer and activation of transcription (STAT)- and MAPK-mediated signaling pathways function downstream of C-KIT [7]. Therefore, we evaluated the effect of celastrol on the expression of these key signaling proteins. The results showed that celastrol significantly suppressed the phosphorylation of AKT, STAT3 and Erk1/2 in Kasumi-1 cells (Figure 5C,D), suggesting these signaling pathways might be associated with celastrol-induced cell death. We next tested the combined effect of celastrol and a AKT inhibitor LY294002 on Kasumi-1 cells, and found that celastrol enhanced the effect of LY294002 on Kasumi-1 cells growth (Figure 5E). To confirm the role of AKT in celastrol-induced cell death, Kaumi-1 cells were transfected with a plasmid bearing a constitutively active form of AKT by electroporation. The growth inhibition by celastrol treatment was partially recovered after transfecting constitutively active AKT plasmid (Figure 5F).

## 3. Discussion

Although the clinical outcomes of AML have markedly improved due to the development of novel therapeutic approaches, long-term survival remains poor, and the rate of disease recurrence remains high. Therefore, there is an urgent need for novel drugs that can prolong survival and improve prognosis in patients with AML. Celastrol exerts potent anticancer effects in various malignancies. In the present study, we demonstrated that celastrol inhibited the growth of t(8:21) AML cells by triggering the extrinsic and intrinsic apoptosis pathways. In addition, celastrol decreased the expression of AML1-ETO and C-KIT in mRNA and protein levels, thereby suppressing the AKT, STAT3 and Erk1/2 downstream signaling pathway.

Apoptosis is a common mechanism used to kill cancer cells. In this study, we observed that the morphological characteristics indicative of apoptosis and DNA fragmentation in cells treated with celastrol (Figure 2A). The Annexin V/PI double staining and DNA ladder assays confirmed that celastrol induced apoptosis (Figure 2B–D). Caspases play essential roles in apoptosis, and the extrinsic and the intrinsic apoptosis pathways are two common apoptotic pathways that lead to the activation of effector caspases [19,20]. Accordingly, caspase activity assays and western blot analysis revealed that celastrol significantly activated caspase-mediated apoptosis pathways (Figure 3A,B). In addition, we observed a partial reduction in celastrol-induced cell death in cells pretreated with caspase inhibitors (Figure 3C–F). These results suggest that caspase activation mediates celastrol-induced apoptosis. The extrinsic pathway, also referred to as the cell death receptor pathway, is initiated by the death receptors (Fas and the TNF receptor) [21].We demonstrated that the expression of Fas, FasL and FADD were upregulated in cells treated with celastrol (Figure 4A), suggesting that the extrinsic apoptosis pathway contributed to celastrol-induced apoptosis. The critical function of the mitochondria in the intrinsic pathway relies on the disruption of the mitochondrial outer membrane permeabilization [22]. MMP inhibition induces the release of Cyt C and other key effector proteins from the mitochondria [23]. We found that celastrol decreased MMP in Kasumi-1 cells (Figure 4B) and enhanced the release of Cyt C and AIF from the mitochondria into the cytoplasm (Figure 4C). In addition, the ratio of Bax: Bcl-2 levels were increased in cells treated with celastrol in a dose-dependent manner (Figure 4D), suggesting that an imbalance between anti-apoptotic proteins and pro-apoptotic proteins is associated with the disruption of MPP. Taken together, celastrol triggers apoptosis via activating the extrinsic and intrinsic apoptosis pathways, consistent with the observation that celastrol activates effector caspases.

AML1-ETO, a chimeric oncogene observed in 20% of patients with AML, plays a well-established role in the leukemogenesis of t(8;21) AML. AML1 (also referred to as RUNX1 or CBFα2) is a transcriptional activator of genes that promote hematopoiesis, and ETO (also referred to as MTG8 or RUNX1T1) functions as a corepressor in protein-protein interactions [5,24]. The AML1-ETO fusion protein recruits ETO-associated repressors, thereby inhibiting the transcription of AML1 target genes and promoting the self-renewal of hematopoietic stem cells. The AML1-ETO fusion protein is considered a therapeutic targets in cancers with the t(8:21) translocation [25]. Previous study demonstrated that silencing of AML1-ETO significantly inhibits cells growth in Kasumi-1 cells [26]. In t(8;21) AML, AML1-ETO upregulates C-KIT and is associated with C-KIT mutation and C-KIT overexpression [6,27]. Gain-of-function mutations in C-KIT and aberrant C-KIT expression have been reported in AML and human gastrointestinal stromal tumors [6,28]. Mutations in *C-KIT* are associated with a poor prognostic and a poor survival rate in AML patients [29,30]. Furthermore, a recent study demonstrated that high levels of C-KIT expression are associated with an increased risk of relapse and a poor prognosis in t(8;21) AML [31]. Therefore, inhibition of AML1-ETO and C-KIT might provide care beneficial for the treatment of t(8;21) AML. In this study, we revealed that celastrol downregulated the AML1-ETO and C-KIT expression at the mRNA and protein levels (Figure 5A,B). These data imply that celastrol may be a promising drug that can target AML1-ETO and C-KIT in the clinical setting [32]. Ligand-induced down-regulation is a key phenomenon that regulates the homeostatic physiology of cell surface receptors. Ligand binding to C-KIT induces receptor dimerization and autophosphorylation at specific tyrosine residues. The activated receptor subsequently phosphorylates proteins to activate signaling cascades that contribute to cell survival, proliferation, differentiation and transformation, such as the pathways mediated by phosphatidylinositol 3-kinase (PI3K)/AKT/mTOR, Ras-Raf-Mitogen-activated protein kinase cascade (MAPK), Janus kinase (JAK)/STAT [33]. Consistent with previous reports, we confirmed that celastrol suppressed the phosphorylation of AKT, STAT3 and Erk in a dose- and time-dependent manner (Figure 5C,D). AKT plays an important role in C-KIT-mediated growth and apoptosis [34]. Combination treatment with AKT inhibitor and celastrol exerted a synergistic effect in inhibiting cell proliferation, while celastrol-induced proliferation inhibition might be partially reversed by constitutively active AKT, suggesting the AKT pathway is associated with loss of C-KIT signaling (Figure 5E,F). Certainly, the role of other kinases in mediating the anticancer activity of celastrol remains to be further investigated. In a previous study, we demonstrated that bortezomib triggered the internalization and degradation of C-KIT, which led to the subsequent release of Apaf-1 and Cyt C, and ultimately resulted in caspase activation [9]. In this study, we revealed that celastrol not only downregulated C-KIT, but also activated the mitochondrial damage response, thereby triggering caspase activation (Figure 2 and Figure 5). Therefore, we conclude that celastrol represents a promising targeted agent for the treatment if AML that induces apoptosis via a C-KIT signaling cascade.

## 4. Materials and Methods

### 4.1. Reagents 

Celatrol was purchased from Calbiochem (San Diego, CA, USA) and dissolved in dimethyl sulfoxide (DMSO) to generate a 50 mM stocking solution and stored at −20 °C until used [14]. Propidium iodide (PI), rhodamine-123 (Rho 123) and β-actin were obtained from Sigma-Aldrich (St. Louis, MO, USA). Antibodies against PARP, Caspase-3, 8, 9, apoptosis-inducing factor (AIF), cytochrome C (Cyt C), Cox-4, phosphor-Erk1/2 (T202/Y204), phosphor-Akt, and phosphor-STAT3 (Y705), STAT3 were obtained from Cell Signaling Technology (CST, Beverly, MA, USA). Enhanced chemiluminescence reagents were purchased from Thermo Fisher Scientific (Waltham, MA, USA).

### 4.2. Cell Culture

The t(8:21)-bearing cells lines, Kasumi-1 and SKNO-1, were obtained from the American Type Culture Collection (Manassas, VA, USA). These cells were cultured in Dulbecco’s RPMI 1640 (Gibco BRL, Grand Island, NY, USA) supplemented with 15% fetal bovine serum (Gibco BRL), 100 U/mL penicillin and 100 U/mL streptomycin as previously descried [35]. Normal human hematopoietic cells were isolated from peripheral blood collected from five volunteer healthy donors using Ficoll density gradient centrifugation. All cells were incubated in a 5% CO_2_ incubator at 37 °C. 

### 4.3. Assessment of Cell Viability, Apoptosis, Cell Morphology and DNA Fragmentation

Cells were treated with celastrol for the indicated concentrations and time points. Cell proliferation was evaluated using Cell Counting Kit-8 (CCK-8) assays (Dojin Laboratories, Kumamoto, Japan), according to the manufacturer instructions. The cell growth curve was estimated using trypan blue dye exclusion assays. Cell morphology was assessed using Wright-Giemsa staining followed by detection using an Olympus microscope (Olympus, Tokyo, Japan). For the DNA fragmentation assays, the cells were collected and lysed. The lysates were incubated with RNase for 30 min at 37 °C, and then incubated with Proteinase K at 50 °C for an additional 16 h. The DNA was subsequently extracted and electrophoresed. The gel was visualized using UV light and images were captured. Cells apoptosis analysis and MPP assays were conducted as previously described [36,37].

### 4.4. Western Blot Assay 

Cells were lysed in RIPA buffer containing 50 mM Tris-HCl (pH 7.4), 150 mM NaCl, 1% Triton X-100, 1% sodium deoxycholate, 0.1% SDS, 1 mM Na_3_VO_4_, NaF 1 mM, a cocktail of 1 mM PMSF and 1 mM protease inhibitors. The lysates were centrifuged at 12,000 g for 10 min at 4 °C, protein concentrations were measured using a spectrophotometer (Thermo Fisher Scientific). The samples were boiled at 100 °C for 7 min, chilled on ice and separated using 10% SDS-PAGE electrophoresis and transferred to nitrocellulose membrane (Millipore Corporation, Billerica, MA, USA) at 100 V for 2 h in 1× transfer buffer. The membrane was blocked in 5% no fat milk/PBST and incubated with primary antibody overnight at 4 °C. β-actin was used as a loading control. The membrane was washed with 0.05% Tween-20/PBS and subsequently incubated with an HRP-conjugated secondary antibody (CST, Beverly, MA, USA), then detected using chemiluminescent substrate (Thermo Fisher Scientific).

### 4.5. Measurement of Mitochondrial Membrane Integrity 

The mitochondrial membrane potential was assayed using rhodamine-123 staining. Briefly, cells were treated with various concentrations of celastrol for the indicated amount of times, collected, washed with PBS and stained with rhodamine-123 for 1 h at 37 °C. The cells were then incubated in PI working solution for 15 min in the dark at RT. The cells were evaluated using flow cytometry analysis.

### 4.6. Preparation of Cell Fractions and Western Blot Analysis

To assess the level of AIF and Cyt C, whole cell lysates were prepared in RIPA buffer supplemented with 0.2 mg/mL digitonin for 5 min at 37 °C. After centrifugation, the supernatant (cytoplasmic fraction) and the pellet (mitochondrial fractions) were separated and evaluated using western blot assays.

### 4.7. Quantitative Real-Time PCR Analysis

Total RNA was extracted from cells using Trizol Reagent (Gibco/BRL). First-strand cDNA was synthesized from 1 μg total RNA using the PrimeScript RT Master Mix (Takara, Tokyo, Japan). The expression of C-KIT mRNA was analyzed using the PrimeScript One Step RT-PCR Kit (Takara), according to manufacturer’s manual. 

### 4.8. Statistical Analysis

The data are presented as mean ± SD. Comparisons between groups were made using ANOVA, and *p* values of 0.05 or less were considered statistically significant.

## 5. Conclusions

In conclusion, the present study reveals that celastrol inhibits t(8;21) leukemia cell proliferation. Our data also indicates that celastrol down-regulates the AML1-ETO/C-KIT proteins and inactivates the downstream signaling pathways. The findings of our present work suggest that celastrol may be an effective chemotherapeutic agent for the treatment of human t(8;21) leukemia.
